# Developing the Diagnostic Adherence to Medication Scale (the DAMS) for use in clinical practice

**DOI:** 10.1186/1472-6963-12-350

**Published:** 2012-10-08

**Authors:** Sara Garfield, Lina Eliasson, Sarah Clifford, Alan Willson, Nick Barber

**Affiliations:** 1 The Centre for Medication Safety and Service Quality, UCL School of pharmacy, Mezzanine Floor, BMA House, Tavistock Square, London, UK; 2 Centre for Haematology, Imperial College London, Hammersmith Hospital, Du Cane Road, London, W12 0NN, UK; 3 National Leadership and Innovation Agency for Healthcare, Bridgend Road, Llanharan, Glamorgan, UK

**Keywords:** Adherence, Compliance, Medication, Medicine, Measure, DAMS, Development, Validity

## Abstract

**Background:**

There is a need for an adherence measure, to monitor adherence services in clinical practice, which can distinguish between different types of non-adherence and measure changes over time. In order to be inclusive of all patients it needs to be able to be administered to both patients and carers and to be suitable for patients taking multiple medications for a range of clinical conditions. A systematic review found that no adherence measure met all these criteria. We therefore wished to develop a theory based adherence scale (the DAMS) and establish its content, face and preliminary construct validity in a primary care population.

**Methods:**

The DAMS (consisting of 6 questions) was developed from theory by a multidisciplinary team and the questions were initially tested in small patient populations. Further to this, patients were recruited when attending a General Practice and interviewed using the DAMS and two other validated self-reported adherence measures, theMorisky-8 and Lu questionnaires. A semi-structured interview was used to explore acceptability and reasons for differences in responses between the DAMS and the other measures. Descriptive data were generated and Spearman rank correlation tests were used to identify associations between the DAMS and the other adherence measures.

**Results:**

One hundred patients completed the DAMS in an average of 1 minute 28 seconds and reported finding it straightforward to complete. An adherence score could not be calculated for the 4(4%) patients only taking ‘when required’ medication. Thirty six(37.5%) of the remaining patients reported some non-adherence. Adherence ratings of the DAMS were significantly associated with levels of self reported adherence on all other measures Spearman Rho 0.348-0.719, (p < 0.01). Differences in trends could generally be explained by qualitative data.

**Conclusion:**

The DAMS has been developed for routine monitoring of adherence in clinical practice. It was acceptable to patients taking single or multiple medication and valid when tested against other adherence measures. However, ‘when required’ medication needs to be excluded. Further tests of the DAMS against objective measures such as MEMS are in progress and reliability needs to be established. Further investigation of the carers’ version of the DAMS is required.

## Background

The Department of Health (DoH) in England has identified the need to build informed, comprehensive, primary care-based medication adherence services which are tailored to patients’ needs [1]. Non-adherence to medication (i.e. patient’s behaviour not matching agreed recommendations from the prescriber) is estimated to affect approximately 30- 50% of patients with chronic conditions [2]. Non adherence may be intentional (i.e patients deciding not to take the medication) or unintentional (patients forgetting or being unable to take their medication). The consequences include a missed opportunity for treatment effect, poor health outcomes and increased healthcare costs. For example, it has been estimated that non-adherence is responsible for 48% of asthma deaths, an 80% increased risk of death in diabetes and a 3.8-fold increased risk of death in the year following a heart attack [3]. In the United Kingdom (UK) National Health Service (NHS), medicines are the biggest expenditure after staff and 71% of the medication budget is spent in primary care (i.e. community settings). It is estimated that the current cost of unused or unwanted medicines exceeds £300 million annually [4]. A recent paper that mapped the quality of medicines use in primary care indicated that an improvement in adherence would be the most significant area to target to improve medicine use [5].

We have identified the need for a self report adherence measure, which can be used to regularly monitor and continuously improve the quality of adherence services [6]. It is a vital tool in applying the Model for Improvement [7] allowing individual clinicians to continuously assess the effects of changes on adherence at individual and population levels.

Self report has been considered the method of choice for measuring non-adherence in clinical practice [8]; it is cheap, relatively unobtrusive, can be used on all types of medicines and is able to distinguish between intentional and unintentional non-adherence (which have different underlying causes and therefore require different interventions). Hence a self report adherence measure used in routine clinical practice is needed, by which adherence could be assessed, an appropriate intervention instigated, and the effectiveness of the intervention evaluated. We think the measure of adherence should have several characteristics. The measure should be rooted in a theory that allows interventions to be tailored towards different types of non-adherence. It would also need be able to be used repeatedly to track patients’ adherence over time and in response to interventions. It should, ideally, be brief and acceptable to patients and be able to be used across a range of clinical conditions. It would need to be able to be completed by or in conjunction with carers where necessary, as it is acknowledged that they can have significant roles in medication management [9]. After conducting a systematic review [6] we were unable to find an adherence measure which met all the above criteria.

### Aim

To develop a theory based adherence scale (the Diagnostic Adherence to Medication Scale-DAMS) and establish its content, face and preliminary construct validity in a primary care population.

## Methods

### Development of DAMS

The Diagnostic Adherence to Medication Scale (DAMS) was developed as a short self-report questionnaire based on patients’ recall of doses missed or doses taken additionally during a 7 day period (see Additional file 1: appendix 1). It is designed to measure adherence for all of patients’ medicines. A team of pharmacists and psychologists were involved in its development. Questions one and two seek to obtain a denominator of the amount of medicine which patients have been prescribed. Question three seeks to obtain the extent of non-adherence to medication (i.e. number of missed doses) and question four seeks to identify the type of non-adherence (intentionally versus unintentionally missing doses). Question five seeks to assess overuse of medication (i.e. taking too many doses) and question six seeks to identify whether this overuse of medication was intentional or unintentional. Adherence levels for the DAMS are calculated based on the amount of medication taken divided by the amount of medication prescribed. These are multiplied by 100 to attain the percentage of doses taken (missed doses would thus provide adherence rate <100% and extra doses taken would provide an adherence rate >100%). If patients are taking more than one medicine the mean adherence level can be calculated to give an overall level. A carers’ version of the DAMS has been developed (see Additional file 1: appendix 2) which can be completed by or in conjunction with carers where necessary.

### Theoretical basis of DAMS

The DAMS is rooted in the accident causation framework [10] (which views non-adherence as predominantly a system issue rather than one of personal blame [11]) and is designed to routinely monitor adherence in clinical practice and to inform the development and evaluation of interventions. Human error theory can be applied to non-adherence, allowing us to establish the underlying factors contributing to individuals not taking medication and enabling us to find appropriate patient centred solutions [11]. Non-adherence can be considered a symptom, rather than a diagnosis and in order to target adherence services to support patients’ needs, it is necessary to diagnose the cause. Intentional and unintentional non-adherence have different underlying causes and require different interventions. Therefore the DAMS has been designed to distinguish between these two types of non-adherence.

We chose to measure adherence as a continuous scale to allow the detection and quantification of changes in adherence levels over time, which will enable use of continuous quality improvement approaches [7] and the evaluation of adherence services and interventions. In addition, a continuous scale can also be converted to a nominal scale to dichotomise adherent and non-adherent patients according to a clinically appropriate predefined cut-off point.

We chose to ask about adherence over the last seven days so that the DAMS could, if necessary, be used repeatedly over relatively short periods of time and measure change in adherence. It was also felt that asking about the last seven days would result in less recall problems than a longer time period.

To reduce the social desirability effect, the questions of the DAMS are framed in a non-judgemental way to assure the patients that non-adherence is common and many patients miss doses at times. The DAMS items therefore use the framing “People often miss taking doses of their medicines, for a whole range of reasons. Thinking of the last 7 days…” before asking about the patient’s own medicine taking behaviour.

In primary care patients are often treated for multiple conditions. Therefore the DAMS is designed to be generic rather than disease specific and to be suitable for patients taking a single medication or multiple medications for different conditions. The DAMS does not include timing errors as exact timing is only important for a small minority of medications.

Ethics approval was obtained from NRES Committee London prior to the commencement of testing of the DAMS.

### Initial testing of the DAMS

During the development process we tested the DAMS on a small convenience sample of patients several times. This was an iterative process of constantly adjusting the wording to make it clearer and gradually extending the sample it was tested on. Following this, to test patient acceptability further we used the DAMS as part of adherence audits in patients taking oral therapy for chronic myeloid leukaemia (n =32 convenience sample during outpatient clinics) and a range of primary care patients (n = 67 random sample) using an existing pharmacy based service designed to help patients with adherence difficulties. The latter sample was mostly elderly. Following these audits further adjustments were made to make the DAMS more user friendly; an additional question for patients who could not remember the names of their medicines was added. At this stage we then wished to test the DAMS in a wider primary care population.

### Testing the DAMS in a wider primary care population

#### Sampling

The population was all patients attending a primary care general practice and their carers where appropriate. The general practice consisted of two centres for health in separate locations which incorporated walk in centres as well as surgeries for regular patients. As this was an exploratory study, a convenience sample of primary care patients was recruited. However, the two centres together serve a diverse range of patients from different socio-economic backgrounds which we believe to be representative of primary care patients.

A sample size of 100 patients was chosen to enable us to explore acceptability and ease of use in a general practice population.

#### Inclusion criteria

Patients attending an appointment at the general practice surgery who were taking at least one prescribed medication. Carers of patients attending appointments at the general practice surgery and using prescribed medication. (All forms of medication were included).

#### Exclusion criteria

Patients not taking medication; patients who were unable to give informed consent, patients not speaking English and children under the age of eighteen.

#### Recruitment

Patients were approached in the waiting room of the general practice surgeries. The researcher explained the study to them and showed them the information leaflet. Patients who wished to participate signed a consent form. This included an additional option for patients to consent to the researcher viewing their medical records. If patients did not have the capacity to consent they were excluded and their carer was asked to participate instead. In these cases the carer signed the consent form and medical records were not viewed.

#### Data Collection

Patients were interviewed in a private room at the general practice. With consent, the interview was audio recorded. The researcher interviewed the patient using the research instruments described below. The questions were read out to the interviewee in relation to all prescribed medication. The order of administering the three scales (described in the next section) was alternated between patients to reduce the potential contamination effect of the order of questions on response. The carer was interviewed where a patient was unable to consent or where they consented but indicated they would prefer the carer to answer the questions. If written consent had been obtained from the patient, the researcher accessed patients’ medical records in order to obtain a list of the name and dosage of medications which had been prescribed.

#### Instruments

We used three adherence instruments: the DAMS, Morisky- 8 [12] and that of Lu et al [13] and also a questionnaire exploring aspects of the acceptability and validity of all the instruments. Selection of these adherence scales followed a systematic review of adherence scales [6] and our rationale was:

1) The DAMS is a new self report measure of adherence which has novel properties that support the finding of practical solutions to non-adherence (see the development section above).

2) The Morisky Eight Item Medication Adherence Scale (Morisky-8) is an adherence measure which has been previously validated in over 1,000 patients with hypertension [12] but not with other conditions (see Additional file 1: appendix 3). The first seven items are dichotomous and the last contains a Likert scale. Six of the eight items address general adherence rather than over a specific time scale and items two and five address adherence over a fortnight and a day respectively. The Morisky-8 produces an overall adherence score which ranges from 1-8, a higher score indicating a greater extent of adherence. Respondents can also be classified as low (score 1-5), medium (score 6-7) or high (score 8) adherers according to their overall score. The Morisky-8 instrument was excluded if the carer rather than patient took part in the interview, as no carers’ version is available. We chose this instrument as it has been developed from a widely used and validated four item scale [14] as well as being validated itself in a large sample.

3) Lu et al [13] designed an adherence questionnaire in which the previous month’s adherence was asked about in three different ways: frequency (eg none of the time; a little of the time;… all the time),percentage (0%; 10%; 20% … 100%) and rating their ability to take their medication as prescribed (very poor; poor; … excellent) (see Additional file 1: appendix 4). The questionnaire has been previously validated in 156 HIV positive patients. In this study we asked about medication use in the last 7 days rather than the last month so that the responses could be more easily compared with those of the DAMS, although we recognise that altering the measure in this way may have affected its psychometric properties. We chose this measure as it includes different question types which have been shown to have different levels of validity and it therefore provided an interesting basis of comparison with the DAMS.

A semi-structured interview addressed the acceptability, face and content validity by asking patients or carers for feedback on acceptability of the measures and ease of understanding. Construct validity was also assessed by exploring reasons for differences in the responses to the three measures. These included direct questions about the differences in responses as well as indirect questions further exploring patient’s medication taking behaviour.

### Analysis

Data was entered onto Statistical Package for the Social Sciences version 15 (SPSS). We investigated acceptability by identifying time to completion, missing data and patients’ qualitative comments about the instruments. We used Spearman rank correlation tests to compare the extent of adherence between the DAMS and the other adherence measures. We then investigated differences in trends between the three instruments and used the qualitative responses patients gave to the questions to understand these differences further using content analysis.

## Results and discussion

### Sample and response

**Figure 1:**
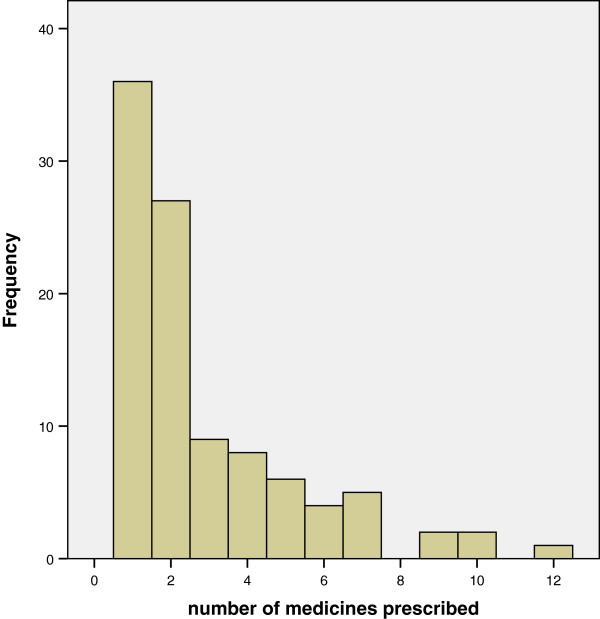
Number of Medicines that respondents were taking.

One hundred and sixty four (62%) out of 266 individuals approached agreed to take part. Of those, 100 (62%) were currently taking medication or were the carer of the patient currently taking medication. Forty two (42%) were male and 58 (58%) were female. In 95 (95%) cases the patient was interviewed, in four cases (4%) the carer was interviewed and in one case (1%) both the patient and carer were interviewed. The range of medicines that had been prescribed indicated a range of clinical conditions. The number of medicines reported as being taken by patients is shown in Figure 1.

Of those declining to participate, 59 (58%) gave reasons. The most commonly cited reasons included not having sufficient English to give consent (n = 16, 25%), not wishing to move from the waiting area to another room (n = 9, 15%), being called in for their appointment before being interviewed and leaving the surgery straight after (n = 9, 15%) and not feeling well enough (n = 6, 10%).

### Acceptability

The measures were not time consuming to complete with the DAMS taking a mean of 1 minute 28 seconds to complete, the Morisky a mean of 1 minute 18 seconds and the Lu a mean of 40.2 seconds. There was a significant moderate correlation between the number of medicines being taken and the time taken to complete the DAMS (spearman 0.61, p = 0.01) which suggests it could take longer than the average time to complete for patients on a large number of medicines. Patients generally commented that the questions were straightforward and easy to answer although there were some difficulties with individual questions of each of the instruments. These difficulties and any subsequent missing data varied between instruments and are described below.

DAMS: For four (4%) of patients, we were not able to calculate an adherence score for DAMS because they had only been prescribed ‘when required’ medication; there was therefore no denominator of how much medication they had been prescribed to take. This is a limitation of the DAMS. Questions one and two enabled a denominator to be calculated in every case where regular medication was prescribed. In all cases where patients had missed doses or taken extra medication it was possible to classify them as intentionally or unintentionally non-adherent. Whilst other studies have found that patients find it difficult to report a specific number of doses missed [6], respondents reported finding the DAMS questions easy to complete. As the DAMS carers’ version was only completed by five respondents, limited data is available on its acceptability. However, no missing data was reported and no problems were identified.

Morisky-8: In four cases (4%), Morisky-8 was not relevant, as a carer was the respondent and there is no carers’ version. Excluding these cases, we were able to calculate a Morisky-8 score and assign an adherence category for 83 (86%) of respondents. For the remaining 13 (14%) of respondents this was not possible as one or more items were missing. The overall volume of missing data was fairly consistent across items. Some specific issues were identified with some items which explained some missing data: item four (When you travel or leave home do you sometimes forget to bring along your medication?) was reported to be irrelevant to one respondent as he was on once daily medication and did not travel, one respondent was unable to answer question five (Did you take your medicine yesterday?) as he had taken some but not all his medication the day before, one respondent was unable to answer question six (When you feel like your condition is under control do you sometimes stop taking your medicine?) because he had consulted the doctor about stopping when his condition was under control and was following their advice and one respondent was unable to answer question seven (Taking medication is a real inconvenience for some people. Do you ever feel hassled about sticking to your treatment plan?) because he had only just started the medication. Whilst not a cause of missing data, respondents identified difficulties with other questions during the qualitative part of the interview. Some respondents found it difficult to choose a yes or no response to question one (Do you sometimes forget to take your medication?) if they rarely forgot. Some respondents interpreted question two (People sometimes miss taking their medication for reasons other than forgetting. Over the past two weeks were there any days when you did not take your medicine?) as referring to both intentional and non intentional non-adherence, and some only to intentional. Some interviewees found it difficult to answer question three (Have you ever cut back or stopped taking your medication because you felt worse when you took it?) as they reported they had stopped medication and then told the doctor at the next appointment. For question six (When you feel like your condition is under control do you sometimes stop taking your medication?) some respondents reported that they stopped taking medication when their condition was under control as it was a ‘when required’ medication. This led to false positive results.

Lu: The Lu questionnaire [13] produces three separate scores for frequency, percentage and rating. Nine (9%) of patients had missing data for the frequency and nine (9%) for rating but thirty five (35%) had missing data for percentages. This large amount of missing data for percentages was explained by the qualitative data. Many respondents reported finding this question difficult to answer and 35 (35%) were not able to respond with a percentage. Some respondents reported difficulty understanding the frequency question; in some cases this was because they were taking ‘when required’ medication. Some respondents described finding the question rating their ability to take medicines difficult to answer, commenting that it was ambiguous, or asking for clarification. In some cases respondents reported a discrepancy between rating and behaviour. For example some respondents stated that their ability was excellent but that they had decided not to take the medication.

### Verification of self reported prescribed medication with medication records

As the regular GP practice was combined with a walk in clinic, medication records were not available for the majority of patients. Patients’ medication was verified by medication records in 20 (20%) of cases. In ten (50%) of these cases the self reported number and dosage of medication on the DAMS corresponded with medication records. In a further six cases (30%) the medication records corresponded with the DAMS apart from ‘when required’ medication; it is possible that patients were using previous supplies of ‘when required’ medication that did not appear on recent records and that patients did not always state their ‘when required’ medication at interview. Medication prescribed ‘when required’ could not be included in the DAMS adherence rating calculation; therefore in a total of 16 (80%) of cases the adherence score would have been identical whether medication records or self report was used to identify prescribed medication. In five (20%) of cases the number or dose of medication prescribed differed between self report and medication records.

### Reported adherence levels

Self reported adherence levels were examined for each of the three adherence measures.

DAMS: To calculate a DAMS score a denominator of the total amount of medication prescribed to be taken in a week was needed. The two possibilities were either to use medication records or patients’ self report of medication prescribed. As described above, medication records were only available for a small proportion of patients. In addition, we wished to test the DAMS as a complete measure of self reported adherence. Therefore patients’ self report of medication prescribed was used. The resulting mean levels of self reported adherence according to the DAMS are shown in Table 1. Of those reporting missed doses (i.e. less than 100% adherence), ten (28%) reported these were intentionally missed, 25 (69%) reported they were unintentionally missed and one (3%) reported both intentional and unintentional non-adherence.

**Table 1:** Extent of self reported non-adherence according to the DAMS (n = 96, missing data excluded)

**Overall level of reported non-adherence**	**Intentional n (%)**	**Unintentional n (%)**	Total n (%)*
**0**%	n/a	n/a	60 (62.5)
**1-9**%	3 (3.1)	8 (8.3)	11 (11.5)
**10-19**%	2 (2.1)	9 (9.4)	11 (11.5)
**20-49**%	2 (2.1)	5 (5.2)	5 (5.2)
**50-99**%	3 (3.1)	2(2.1)	6 (6.3)
**100**	1 (1.1)	2 (2.1)	3 (3)

*Some patients were both intentionally and unintentionally non-adherent.

Four patients (4%) had been non-adherent by taking extra medication. In three cases (75%) this was reported to be intentional and in the fourth case (25%) both intentional and unintentional non-adherence were reported.

Morisky-8: The total Morisky-8 score ranged from 1 to 8 with the mean score being 5.86 (standard deviation 1.82). The number of patients classified as high, medium and low adherers are shown in Table 2.

**Table 2:** Classification of adherence according to Morisky-8 (n = 83, missing data excluded)

**Adherence category**	**n (%)**
High	21 (25)
Medium	26 (31)
Low	36 (44)

Lu: The vast majority of respondents reported taking medication most or all of the time (See Table 3), taking their medication over 80% of the time (see Table 4) and that their ability to take their medication was good-excellent (Table 5).

**Table 3:** Frequency of adherence according to Lu Questionnaire (n = 87 missing data excluded)

**Frequency of medication adherence in last 7 days**	**n (%)**
None of the time	4 (4.6)
A little of the time	3 (3.4)
Some of the time	3 (3.4)
A good bit of the time	1 (1.1)
Most of the time	27 (31)
All of the time	49 (56.5)

**Table 4:** % of adherence according to Lu questionnaire(n = 65 missing data excluded)

**% adherence**	**n(%)**
0	2 (3.1)
10	1 (1.6)
20	0 (0)
30	1 (1.6)
40	0 (0)
50	1 (1.6)
60	1 (1.6)
70	5 (7.8)
80	6 (9.4)
90	13 (20.3)
100	34 (53)

**Table 5:** Rating of adherence according to Lu questionnaire (n = 91 missing data excluded)

**Adherence rating**	**n(%)**
Very poor	2 (2.3)
Poor	2 (2.3)
Fair	4 (4.6)
Good	19 (21.8)
Very good	24 (27.6)
Excellent	36 (41.4)

Tables 1, 3 and 4 also demonstrate that the DAMS, LU frequency and Lu percentage were able to detect 0% adherence. Patients identified that they had been prescribed medication but had not taken any in the last 7 days. In the semi structured interviews patients clarified that they had either stopped the medication, decided to take a drug holiday or forgotten all doses in the last 7 days. (The Morisky-8 and Lu rating are not designed to distinguish between low and zero adherence).

### Comparisons between measures

Table 6 shows the results of the Spearman rank correlation tests to compare adherence on the DAMS to that reported on the other adherence measures. As shown in the table, adherence ratings of the DAMS were moderately to strongly correlated with self reported adherence on all other measures at the p = 0.01 level.

**Table 6:** Associations between adherence measures

**Instrument**	**Spearman’s Rho**	**P value**
Morisky-8 and DAMS	0.472	<0.001
Lu frequency and DAMS	0.719	<0.001
Lu percentage and DAMS	0.546	<0.001
Lu rating and DAMS	0.348	0.001

**Figure 2:**
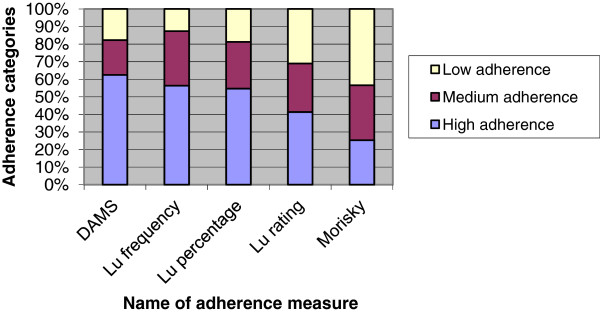
Comparison of adherence measures.

Figure 2 further summarises the adherence levels obtained by the different adherence measures to allow comparisons to be made. Whilst only the authors of Morisky-8 have classified adherence as high, medium or low; in order to allow easier comparisons between instruments, for the purposes of this figure only, adherence on all measures was classified as high, medium or low. When developing this classification for the DAMS and Lu we followed the Morisky-8 classification. High was equal to perfect adherence (100%, excellent, taking medication all the time), medium was very good but not perfect adherence (80-< 100%, taking medication most of the time) and all other levels of adherence were classified as low.

Figure 2 shows that although there were significant associations between the DAMS and other measures, there was a trend, when compared to the DAMS, for higher non-adherence to be reported on the Lu questionnaire and still higher non-adherence to be reported on the Morisky- 8 scale. Below we use the findings of the semi-structured interviews to explain this trend.

Morisky-8 vs DAMS: The majority of differences between Morisky-8 and DAMS could be explained by there having been differing adherence levels at different points in time. The DAMS asks about adherence in the last seven days whereas one item of the Morisky-8 asks about the previous day, one item asks about the previous 14 days and the remaining six items do not specify a time period.

This explanation of differing adherence levels at different points of time was initially seen when analysing low adherers on the Morisky-8. Whilst respondents reporting no missed doses on DAMS were more likely to be high or medium adherers than low adherers on Morisky-8, 13 (36%) respondents classified as low adherers on Morisky-8 reported no missed doses on the DAMS. Seven (54%) of these cases can be fully explained by the fact that the majority of Morisky-8 questions do not ask about a specific time period, whereas the DAMS specifically asks about the last seven days. Therefore respondents reported that they had exhibited some non-adherent behaviours at some point in their lives but not in the previous seven days. In four further cases (31%) patients had been non-adherent in the last 14 days and therefore responded positively to question two of the Morisky-8 (People sometimes miss taking their medication for reasons other than forgetting. Over the past two weeks were there any days when you did not take your medicine?) but had not been non-adherent in the last seven days and therefore did not report non adherence on the DAMS. In three cases (21%) respondents were able to explain this change in recent adherence due to having been unwell or on holiday. In one case (8%) an interviewee responded that they had not taken their medication yesterday on question five of the Morisky-8 as it was not prescribed to be taken every day.

Due to the trend of differing adherence at different time periods observed with low adherers, a further analysis was done of all respondents reporting that they had missed doses in the last 14 days or yesterday on Morisky-8 and reporting no missed doses on DAMS. In all three cases where respondents reported that they had not taken their medication yesterday (on the Morisky-8) but had not missed any doses (on the DAMS) the medication had not been prescribed to be taken on the previous day, either because it was a weekly medication or because the course had been completed. Only six respondents had missed doses in the last 14 days (Morisky-8) but had not missed any in the last seven days (DAMS) and in four of these cases, the respondent was able to offer a reason for the difference in adherence.

Whilst most of the differences between the Morisky-8 and DAMS could be explained by time period some other factors were observed. Four high adherers on Morisky-8 reported missed doses on DAMS. In two cases this could be explained by the fact that Morisky-8 asks about ‘your medication’ rather than ‘all your medication’ so respondents said that they had taken their medication the previous day even though they had missed some of their medication(s). The time at which medication had been taken was another cause of difference in reported adherence, with some patients taking their medication at a later time (which is usually not critical for effectiveness) and reporting this on either scale as a missed or forgotten dose.

Lu is compared to DAMS in three ways, according to the reporting method.

Lu frequency vs DAMS: Eight of the one hundred respondents reported being fully adherent on the DAMS but taking their medication less than all the time in the last seven days on the Lu. Conversely two respondents reported missing doses on DAMS but taking their medication all the time in the last seven days on Lu. Four of these discrepancies could be explained. In two of the ten cases where there were discrepancies these were due to patients taking ‘when required’ medication. The LU does not distinguish between different medicines and therefore patients on a mixture of ‘when required’ and regular medicine did not state that they had taken all their medication all the time if they had only taken the ‘when required’ medicine occasionally. In one case a respondent had only just been prescribed the medication so had not taken it all the time in the last seven days but had not missed a dose and one respondent had insufficient English to understand the frequency question.

Lu percentage vs DAMS: Eight (8%) respondents reported being fully adherent on the DAMS but being able to take their medication less than 100% of the time exactly as their doctors had prescribed according to Lu. In five cases this was due to incorrect timing and in one case the respondent had only been prescribed the medication from the day before. In two cases there was no explanation for the discrepancy. Four respondents reported missing doses on the DAMS but taking their medication as prescribed 100% of the time on the Lu. In two cases the discrepancy was due to timing errors and in one case the patient had missed doses when he had wanted to drink alcohol on Saturday but his doctor had said it was ok to miss doses in these circumstances so he had in essence still taken his medication as his doctor had prescribed 100% of the time. In the fourth case no explanation was given.

Lu rating vs DAMS: In 24/100 cases respondents rated their ability to take their medication as less than excellent but reported missing no doses according to the DAMS and in only six cases respondents rated their ability as excellent but reported that they had missed doses. This is an interesting finding as Lu et al found that the rating question more closely resembled ‘objective’ measures than the frequency and percentage measures. It is not clear why patients rate their ability to take medication lower than their actual medication behaviour and an explanation was only offered by five (19%) respondents. For example one respondent had not missed any doses but rated his ability as good because he finds inhaler technique difficult. However, as noted above this question was thought to be unclear by many respondents and it also emerged that different respondents interpreted the question differently. The rating question is asking for a value judgement rather than actual medication taking behaviour and respondents may avoid extremes and rating themselves as excellent.

### Limitations

Several limitations need to be acknowledged. This study has focused on establishing face and content validity of the DAMS and preliminary construct validity when compared to other self report measures. However, further testing is needed to establish validity against other measures, such as the Medication Event Monitoring System (MEMS) , where the medication lid contains a microchip which records every opening of the medicine container. Further validation of the DAMS is underway including the use of the DAMS in a large clinical trial. In addition, reliability of the DAMs needs to be established.

We changed the time period over which adherence was measured in the Lu questionnaire. Whilst this may have affected the psychometric properties of the Lu scale, we were not using it as a gold standard measure here. Rather, we wished to explore differences between different question types and response options; and the Lu questionnaire with the three different response options was a useful comparator.

The comparison of patient medication records against self reported medication history on the DAMS could only be established in a small number of patients (n = 16), which was a limitation to this part of the analysis.

We recruited patients who were attending appointments at the primary care practice which meant that housebound patients needing home visits would not have been included in this study. However face and content validity of the DAMS has previously been established in a population of patients using a pharmacy service designed for people with adherence problems, many of whom were housebound.

## Conclusion

Adherence levels captured by the three different adherence instruments are in agreement when measuring patients’ medication taking behaviour in a primary care population of patients attending general practice. All struggled with ‘as required’ medication which we think should be excluded from these measures. On further analysis, differences in trends between the DAMS and the other two adherence measures could mostly be explained by qualitative data, although the Lu rating question needs further clarification. The association of the DAMS with two validated adherence instruments provides some validation for the new instrument. The DAMS was developed specifically to meet the criteria for routinely monitoring adherence in clinical practice, as well as supporting the development and evaluation of adherence enhancing interventions. It was able to measure type and extent of non-adherence thereby aiding in the identification of appropriate interventions and measuring change in adherence over time; it also has the advantage of having a carers’ version. In summary, the DAMS has now been tested in a range of populations including housebound patients, patients attending general practice appointments and a secondary care population. Further tests of the DAMS against objective measures such as MEMS are in progress and reliability needs to be established. Testing of the carers’ version of the DAMS is at the preliminary stage and further investigation is required.

## Abbreviations

DAMS: Diagnostic Adherence to Medicines Scale; MEMS: Medication Event Monitoring System; UK: United Kingdom; NHS: National Health Service; DoH: Department of Health; Morisky-8: Morisky Eight Item Medication Adherence Scale.

## Competing interests

The authors declare that they have no competing interests.

## Authors’ contributions

SG was involved in the conceptualisation, development and initial testing of the DAMS, conducted the study and wrote the paper. LE was involved in the conceptualisation, development and initial testing of the DAMS, statistical testing and editing the paper. SC was involved in the conceptualisation, development and initial testing of the DAMS. AW was involved in the conceptualisation of the DAMS and editing of the paper. NB was involved in the conceptualisation and development of the DAMS and editing the paper. All authors read and approved the final manuscript.

## Authors’ information

SG is a postdoctoral researcher at The School of Pharmacy, University College London whose research interests include adherence, concordance and medicines safety. LE was a research associate at Imperial College London, at the time this research was designed and conducted. Her research interests include medicine adherence in chronic illness, use of oral oncology drugs and behavioural change interventions. She is currently Lead Health Psychology Specialist at Atlantis Healthcare.

SC was a Lecturer at The School of Pharmacy, University College London, at the time this research was designed and conducted. She is now a Research Scientist at UnitedBioSource Corporation, Bethesda, MD in the USA.

AW is joint director of the 1000 Lives Plus programme, a Director of the National Leadership and Innovation Agency in Wales, Honorary Senior Lecturer in Swansea University, an Associate of the Welsh Institute for Health and Social Care at the University of Glamorgan and Visiting Professor at the School of Pharmacy, University College London. He was recently made a fellow of the Royal Pharmaceutical Society.

NB is Professor in the Department of Practice and Policy at the School of Pharmacy, University College London, and a Visiting Professor in Patient Safety at Harvard Medical School. He is director of research and education at The Health Foundation.

## Pre-publication history

The pre-publication history for this paper can be accessed here:

http://www.biomedcentral.com/1472-6963/12/350/prepub

## Supplementary Material

Additional file 1 Appendix. Click here for file
