# Supplementary material for: Developing the Diagnostic Adherence to Medication Scale (the DAMS) for use in clinical practice
Source: BMC Health Serv Res. 2012 Oct 8;12:350. doi: 10.1186/1472-6963-12-350 (PMC3507707; doi:10.1186/1472-6963-12-350)
Supplement: Additional file 1 — Appendix. [file 1472-6963-12-350-S1.doc]

Appendix 1 DAMS

These questions ask about how you are getting on with taking your medicines. We know that many patients at times miss or change doses of their medicines. Some forget, others have various problems taking their medicine, and yet other patients adapt their treatment so it better fits in with their life. Please answer the following questions the best you can. The information will help us to understand how we can better adjust individual patients’ treatments to suit their life.

**1) Thinking of the medicines your doctor has prescribed:**

A) WHICH medicines have you been asked to take?

B) How MUCH of these MEDICINES have you been asked to take each DAY:

*Record answers on response sheet; DAMS page 3 – question 1A and question 1B*

**If patient is able to answer and taking medication go to question 3**

**If patient is not taking medication exclude.**

**If s/he is not sure go to question 2**

**2 Thinking of the medicine your doctor has prescribed:**

A) How many tablets and capsules have you been asked to take each day?

B) How many spoonfuls of medicine have you been asked to take each day?

C) How many puffs of inhalers have you been asked to take each day?.

D) How many creams have you been asked to use each day?

E) How many injections have you been asked to have each day?

F) Have you been asked to use any other type of medication?

If so how much?

*Record answers on response sheet; DAMS page 4 – question 2*

**3) People often miss taking their medicines, for a whole range of reasons. Thinking of the last 7 days:**

A) HOW MUCH of each medicine have you MISSED taking in the last 7 DAYS?

*Record answers on response sheet; DAMS page 3 – question 3*

**If patient has NOT missed any, GO TO question 5**

**4) Here are examples of reasons other people have given for missing medicines. THINKING OF THE MEDICINE YOU MISSED IN THE LAST 7 DAYS, which of the following statements best describe what happened (you can choose more than one option)?**

A) I decided not to take it 🗆

B) I forgot to take it 🗆

C) I was unable to take it 🗆

D) Other (please specify) ……………………………………………………………………………

**5) People often take more of their medicine than has been prescribed. Thinking of the last 7 days:**

A) How MUCH EXTRA of each medicine did you take in the last 7 DAYS?

*Record answers on response sheet; DAMS page 3 – question 5*

**If patient has taken extra GO TO Question 6**

**6) Here are examples of reasons other people have given taking extra medicine. THINKING OF THE EXTRA MEDICINE YOU HAVE TAKEN IN THE LAST 7 DAYS, which of the following statements best describe what happened (you can choose more than one option)?**

A) I decided to take more 🗆

B) I accidentally took more 🗆

C) Other (please specify) …………………………………………………………………………………………………………………

Page 3: Response sheet for DAMS questions 1A, 1B, 2, 3A and 5A

| ***Question 1A*** | ***Question 2*** | ***Question 1B/2*** | ***Question 3*** | ***Question 5*** |
| --- | --- | --- | --- | --- |
| **Name of medicine** | **Dosage form** | **Amount PRESCRIBED** | **Amount MISSED in last 7 DAYS** | **Amount EXTRA in last 7 DAYS** |
| **Med 1**  **....................................................................** | **Tablets/capsules** |  |  |  |
| **Med 2**  **....................................................................** | **Liquids** |  |  |  |
| **Med 3**  **....................................................................** | **Inhalers** |  |  |  |
| **Med 4**  **....................................................................** | **Creams** |  |  |  |
| **Med 5**  **....................................................................** | **Injections** |  |  |  |
| **Med 6**  **....................................................................** | **Other**  **..........................** |  |  |  |
| **Med 7**  **....................................................................** | **Other**  **..........................** |  |  |  |
| **Med 8**  **....................................................................** | **Other**  **..........................** |  |  |  |
| **Med 9**  **....................................................................** | **Other**  **..........................** |  |  |  |
| **Med 10**  **....................................................................** | **Other**  **.........................** |  |  |  |

**NB Fill in either question 1A or Question 2**

Appendix 2 DAMS Carers

These questions ask about how [name of patient] is getting on with taking his/her medicines. We know that many patients at times miss or change doses of their medicines. Some forget, others have various problems taking their medicine, and yet other patients adapt their treatment so it better fits in with their life. Please answer the following questions the best you can. The information will help us to understand how we can better adjust individual patients’ treatments to suit their life.

**1) Thinking of the medicines [name of patient]’s doctor has prescribed:**

A) WHICH medicines has [name of patient] been asked to take?

B) HOW MUCH of these MEDICINES has s/he been asked to take each DAY:

*Record answers on response sheet; DAMS page 3 – question 1A and question 1B*

**If carer is able to answer and the patient is taking medication go to question 3**

**If the carer reports that the patient is not taking medication exclude.**

**If s/he is not sure go to question 2**

**2 Thinking of the medicine the doctor has prescribed:**

A) How many tablets and capsules has s/he been asked to take each day?

B) How many spoonfuls of medicine has s/he been asked to take each day?

C) How many puffs of inhalers has s/he been asked to take each day?.

D) How many creams has s/he been asked to use each day?

E) How many injections has s/he been asked to have each day?

F) Has s/he been asked to use any other type of medication?

If so how much?

*Record answers on response sheet; DAMS page 3 – question 2*

**3) People often miss taking their medicines, for a whole range of reasons. Thinking of the last 7 days:**

A) HOW MUCH of each medicine has s/he MISSED taking in the last 7 DAYS?

*Record answers on response sheet; DAMS page 3 – question 3*

**If patient has NOT missed any, GO TO question 5**

**4) Here are examples of reasons other people have given for missing medicines. THINKING OF THE MEDICINE s/he MISSED IN THE LAST 7 DAYS, which of the following statements best describe what happened (you can choose more than one option)?**

A)I decided not to give it to him/her 🗆

B)S/he decided not to take it 🗆

C) I forgot to give it to him/her 🗆

D) S/he forgot to take it 🗆

E) I was unable to give it to him/her 🗆

F) S/he was unable to take it 🗆

G) Other (please specify) ……………………………………………………………………………

**5) People often take more of their medicine than has been prescribed. Thinking of the last 7 days:**

A) How MUCH EXTRA of each medicine did s/he take in the last 7 DAYS?

*Record answers on response sheet; DAMS page 3 – question 5*

**If patient has taken extra GO TO Question 6**

**6) Here are examples of reasons other people have given taking extra medicine. THINKING OF THE EXTRA MEDICINE S/HE HAS TAKEN IN THE LAST 7 DAYS, which of the following statements best describe what happened (you can choose more than one option)?**

A) S/he decided to take more 🗆

B) I decided to give him/her more 🗆 C) S/he accidentally took more 🗆

D) I accidently gave her more 🗆

E) Other (please specify) …………………………………………………………………………………………………………………

Page 3: Response sheet for DAMS questions 1A, 1B, 2, 3A and 5A

| ***Question 1A*** | ***Question 2*** | ***Question 1B/2*** | ***Question 3*** | ***Question 5*** |
| --- | --- | --- | --- | --- |
| **Name of medicine** | **Dosage form** | **Amount PRESCRIBED** | **Amount MISSED in last 7 DAYS** | **Amount EXTRA in last 7 DAYS** |
| **Med 1**  **....................................................................** | **Tablets/capsules** |  |  |  |
| **Med 2**  **....................................................................** | **Liquids** |  |  |  |
| **Med 3**  **....................................................................** | **Inhalers** |  |  |  |
| **Med 4**  **....................................................................** | **Creams** |  |  |  |
| **Med 5**  **....................................................................** | **Injections** |  |  |  |
| **Med 6**  **....................................................................** | **Other**  **..........................** |  |  |  |
| **Med 7**  **....................................................................** | **Other**  **..........................** |  |  |  |
| **Med 8**  **....................................................................** | **Other**  **..........................** |  |  |  |
| **Med 9**  **....................................................................** | **Other**  **..........................** |  |  |  |
| **Med 10**  **....................................................................** | **Other**  **.........................** |  |  |  |

**NB Fill in either question 1A or Question 2**

**Appendix 3 Morisky eight item medication adherence scale**

| **You indicated that you are taking medication for your (identify health concern, such as “high blood pressure”). Individuals have identified several issues regarding their medication-taking behavior and we are interested in your experiences. There is no right or wrong answer. Please answer each question based on your personal experience with your [health concern] medication.** | | |
| --- | --- | --- |
| **(Please cross the given answer)** | | |
|  | **No=0** | **Yes=1** |
| 1. Do you sometimes forget to take your [health concern] medication?…...  (unintentional) |  |  |
| 1. People sometimes miss taking their medications for reasons other than forgetting. Thinking over the past two weeks, were there any days when you did not take your [health concern] medication?…...   (intentional) |  |  |
| 1. Have you ever cut back or stopped taking your [health concern] medication without telling your doctor, because you felt worse when you took it?……...   (intentional) |  |  |
| 1. When you travel or leave home, do you sometimes forget to bring along your [health concern] medication?………………………...   (unintentional) |  |  |
| 1. Did you take your [health concern] medicine yesterday?………..   (unintentional) |  |  |
| 1. When you feel like your [health concern] is under control, do you sometimes stop taking your medicine?………………………………   (intentional) |  |  |
| 1. Taking medication everyday is a real inconvenience for some people. Do you ever feel hassled about sticking to your [health concern] treatment plan?…………………………………………….   (intentional) |  |  |

8. How often do you have difficulty remembering to take all your medications?

(**Please cross the given answer)**

Never/Rarely……………………………………….**0**

Once in a while…………………………………….**1**

Sometimes………………………………………....**2**

Usually…………………………………………….**3**

All the time………………………………………..**4**

(unintentional)

Appendix 4: Lu

1. **In the last 7 days did you take all your medications:**

None of the time 🗆

A little of the time 🗆

Some of the time 🗆

A good bit of the time 🗆

Most of the time 🗆

All of the time 🗆

2) **What % of the time were you able to take your medicines exactly as your doctor prescribed them in the last 7 days?**

0% 🗆

10% 🗆

20% 🗆

30% 🗆

40% 🗆

50% 🗆

60% 🗆

70% 🗆

80% 🗆

90% 🗆

100% 🗆

3**) Rate your ability to take all your medicines prescribed in the last 7 days?**

Very poor 🗆

Poor 🗆

Fair 🗆

Good 🗆

Very good 🗆

Excellent 🗆
